# *In vitro* models of microbiota-gut-brain axis communication at the blood-brain barrier interface

**DOI:** 10.1177/0271678X261419964

**Published:** 2026-02-16

**Authors:** Maya R Davies, Courtney B Cross, María Rodriguez Aburto, John F Cryan, Hannah R Wardill

**Affiliations:** 1 Faculty of Health and Medical Sciences, School of Biomedicine, University of Adelaide, Adelaide, SA, Australia; 2 Precision Medicine, South Australian Health and Medical Research Institute, Adelaide, SA, Australia; 3 APC Microbiome Ireland, University College Cork, Cork, Ireland; 4 Department of Anatomy and Neuroscience, University College Cork, Cork, Ireland

**Keywords:** Blood–brain barrier, microbiota-gut-brain axis, in vitro, modelling, short chain fatty acids

## Abstract

Microbiota-gut-brain axis dysfunction is increasingly implicated in the development of various neuropathologies. The blood-brain barrier (BBB) serves as a critical interface between the central nervous system (CNS) and the systemic milieu, modulated by the gut microbiota and associated secretome. Increasingly, the therapeutic potential of microbial metabolites, such as short-chain fatty acids (SCFAs), in reducing BBB disruption and mitigating neuropathologies across multiple neurological conditions has been explored. However, research methodologies remain inconsistent, owing to a lack of clarity on how to effectively model microbiota-gut-brain interactions at the BBB interface. In order to fully realise the potential of the microbiota-gut-brain axis it is crucial to adopt best-practice study designs alongside ongoing advancements in biotechnology that enable more biologically relevant modelling of this complex system. In this review, we examine current knowledge on the role of the BBB in mediating microbiota-gut-brain interactions and explore both established and emerging methods used to study these processes.

## Background

### Gut microbiota and the microbiota-gut-brain axis

The gut microbiota has elicited wide attention from the medical field and the general public over the previous decades, with extensive scientific findings establishing that the trillions of microbes lining our gastrointestinal tract have the capacity to influence our behaviour and physiology.^1–3^ Such discoveries have informed and reshaped how we understand health and disease, with a potential role of the microbiota in both disease aetiology and treatment. As a result, a variety of gut microbiota-directed therapies have emerged for a range of diseases associated with disruption of the gut microbiota, such as the use of faecal microbiota transplantation (FMT) for *Clostridium difficile* infection management.^4^ However, it has also become clear that the influence of the gut microbiota is not restricted to exerting localised effects in the gastrointestinal (GI) microenvironment, with evidence of a role in most, if not all, organ systems from immune to cardiovascular.^5–7^ The neuroscience field has been far from exempt from this excitement, with an emerging role of the gut microbiota in a number of neurological diseases across the human lifespan, from developmental disorders during childhood, such as autism spectrum disorder (ASD), to neurodegenerative disorders, such as Parkinson’s and Alzheimer’s diseases, in old age.^8–10^ In fact, the presence of the toxic aggregates which drive Parkinson’s disease pathology – alpha (α)-synuclein – can be observed within the GI tract prior to their accumulation in the central system, paralleling the prodromal, clinical GI symptoms which predate the hallmark motor deficits.^1^

With this emerging evidence, comes the accompanying question; how is the gut microbiota influencing the brain, the organ located the greatest distance from the colon. Four concurrent pathways have been identified: (i) the neural pathway, including the vagal nerve (most direct pathway between the gut and the brain) and the enteric nervous system (branch of the peripheral nervous system interacting directly with the gut microbiota), (ii) the immune pathway – with the GI tract the largest immunological organ in the body, based on the density of immune cells, which are constantly interacting with the microbes it hosts, (iii) the endocrine pathway – via modulating gut hormones and the hypothalamic-pituitary-adrenal axis and (iv) the microbial pathway – via secreted metabolites and products within the bloodstream.^2^ In fact, the overall predominant method of microbiota-host communication is thought to be the metabolic pathway, with microbial metabolites (i.e. secretome) exerting significant local and widespread influence.^12^ Microbial metabolites are synthesised via bacterial metabolism of dietary material within the GI tract, and these metabolites can then enter systemic circulation via permeating the gut epithelial barrier.^13^ This translocation may occur via passive diffusion, active diffusion or even leakage when the gut barrier is compromised. Once in circulation, in order for these microbial metabolites to influence the brain, they must first interact with the blood-brain barrier (BBB)^12^ – the specialised endothelial layer that regulates systemic-to-central communication. As such, efforts have turned to modelling and evaluating the BBB, to define its role in microbiota-gut-brain communication. However, this is a rapidly evolving field with a magnitude of methodological strategies that can be adopted, with varying advantages and limitations. This review seeks to provide an overview of *in vitro* preclinical methods, with the goal of improving the rigor and consistency by which BBB-microbiota interactions are assessed in the laboratory environment and accelerate translational success.

### Blood-brain barrier

**Figure 1.**
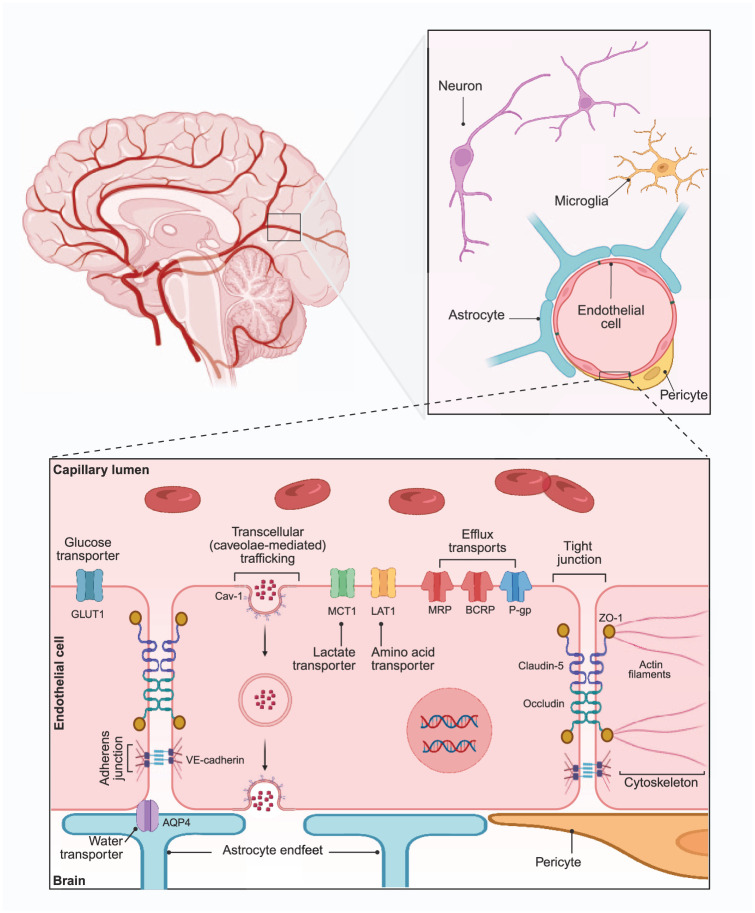
Structure of the BBB including key BMEC cellular structures and components of the NVU. Original figure created with Biorender.com.

The BBB is a multi-cellular structure, with a highly specified environment, which functions to protect the vulnerable brain tissue from potentially harmful molecules within systemic circulation (Figure 1). A monolayer of specialised endothelial cells, brain microvascular endothelial cells (BMECs), comprise the brain capillaries – the main structural component of the BBB.^14^ These BMECs tightly regulate the entry and efflux of molecules to and from the brain, in order to maintain brain homeostasis and meet energy demands. To perform this function, BMECs highly express intercellular tight and adherens junctions to restrict paracellular permeability and also a number of efflux transporters to remove toxins.^14^ Numerous other cells form the BBB and neurovascular unit (NVU) to assist BMECs to maintain this regulation.^14^ These include (i) pericytes (specialised mural cells) which help to regulate capillary diameter and promote endothelial function via both structural and chemical means,^15^ (ii) astrocytes, with astrocytic end-feet in direct contact with BMECs to exert paracrine influence and expression of aquaporin-4 (AQP4) facilitating water transport, (iii) microglia, the resident immunocompetent cells of the central nervous system (CNS) which modulate BBB permeability via secretion of pro- and anti-inflammatory cytokines^16^ and (iv) neurons, with emerging evidence that neuronal activity can, in fact, directly regulate BBB permeability.^17^

Disruption of the BBB is an aetiological factor shared by a number of neurological and neurodegenerative diseases such as ASD, multiple sclerosis, ischaemic stroke, Alzheimer’s and Parkinson’s diseases and CNS tumour.^18–20^ Although the BBB is highly impermeable under normal physiological conditions, permeability can vary between brain regions and over time, and may be altered by developmental stage, ageing or disease.^19,21^ It is becoming evident that even relatively subtle increases in permeability (i.e. leakage) can have quite detrimental impacts. This leakage could be paracellular, with changes in tight and/or adherens junction complexes leading to unregulated influx of peripheral factors (such as pro-inflammatory cytokines, reactive oxygen species or neuroimmune cells) to the CNS or also transcellular, via upregulation of caveolae or downregulation of efflux transporters.^22^ Whatever the means, this leakage results in disruption and heightened exposure of the vulnerable neuronal microenvironment. As such, pathways by which the gut microbiota may modulate BBB permeability have been investigated to understand the role of the gut-brain axis in neuropathologies with greater clarity.

### Gut microbiota-BBB interactions

The principal evidence illustrating the fundamental connection between the gut microbiota and the BBB stems from experimental work using germ-free (GF) rodent models. GF animals are devoid of a gut microbiota from birth and are raised in sterile conditions to maintain this condition.^24^ Notably, the BBB of GF mice is more permeable than their commensally colonised, specific-pathogen free (SPF) counterparts.^25^ This heightened leakage has been linked to reduced expression of key proteins forming the intercellular tight junctions between BMECs, claudin-5 and occludin,^25^ highlighting a structural deficit alongside functional changes. The ability to reverse both the reduction in tight junction proteins and the heightened barrier permeability by delivering the faecal microbiota of normal SPF mice, suggests that a functional BBB requires the presence of a gut microbiota.^25^ However, comparison of the presence/absence of a gut microbiota does lack the finesse to be truly clinically relevant, and therefore the use of antibiotics to disrupt the microbiota provides further insight. Oral delivery of antibiotics (amoxicillin-clavulanic acid) to rhesus monkeys reduced the diversity of microbial families which correlated with increased BBB permeability (determined by increased entry of plasma protein albumin to the CNS).^26^ Of particular interest were reductions in relative abundance of certain microbial genera (all from the Firmicutes phylum, including *Faecalibacterium*, *Blautia*, *Ruminococcus*) which are linked to the production of important microbial metabolites – short chain fatty acids (SCFAs).^26^ Levels of these SCFAs within rhesus monkeys’ faecal contents were negatively correlated with BBB permeability in thalamic and hippocampal brain regions.^26^

SCFAs have generated increasing interest in recent years and now are perhaps the most extensively researched gut microbial metabolites – certainly in the context of the gut-brain axis. SCFAs are produced through the process of complex, plant-derived polysaccharide anaerobic fermentation by bacteria residing within the colon. The three most common SCFAs are acetate, propionate and butyrate, and the absence of these in GF mice is considered to be a key player in associated BBB dysfunction.^25,27^ Both colonisation of GF mice with single microbial strains associated with high SCFA production (*Clostridium tyrobutyricum* or *Bacteroides thetaiotaomicron*) and oral delivery of butyrate alleviated the BBB leakage (defined by albumin CNS extravasation) inherent to GF mice, and direct butyrate delivery increased levels of occludin expression in the frontal cortex and hippocampus.^25^ The influence of SCFAs on the BBB is not restricted to GF animals; oral intake of sodium propionate and sodium butyrate demonstrated beneficial effects in a mouse model of Alzheimer’s disease (*App*^NL-G-F^), by improving tight junction structure, reducing neuroinflammation and partially alleviating toxic amyloid-β plaque accumulation.^27^ Similarly, a role of the gut-microbiota secretome in outcomes of ischaemic stroke has been demonstrated pre-clinically, with reduced levels of a serum biomarker of BBB damage alongside decreases in infarct area and overall reduced neurobehavioural impairments when butyrate was delivered following the stroke.^28^ Stress has been shown to reduce levels of SCFAs, alter BBB function and affect hippocampal plasticity all of which can be countered by exogenous SCFA administration.^29,30^ Further evidence of multiple levels of pathology-altering effects induced by SCFAs has also been identified in preclinical models of Parkinson’s disease,^31–33^ suggesting that these microbial metabolites have the capacity to not only improve BBB function but also other disease-driving neuropathologies simultaneously.

The development of *in vitro* models of the BBB has advanced our ability to dissect causal mechanisms and implicated pathways by which systemic mediators influence cerebral endothelium. BBB leakage in these models is often induced via exposure to endotoxin (lipopolysaccharide (LPS)), a component of the cellular membrane of Gram-negative bacteria and often associated with a disrupted or pathogenic gut microbiota.^34^ LPS treatment deleteriously impacts BMEC expression of tight junction proteins (claudin-5, occludin, ZO-1) and increases barrier permeability (trans-endothelial electrical resistance, TEER) *in vitro*.^12,35,36^ This disruption appears to utilise JNK and p38MAPK mediated pathways and matrix metalloproteinase (MMP-2, MMP-9) signalling as inhibition of these enzymes can partially alleviate LPS-induced tight junction downregulation.^36^ Given their *in vivo* barriergenic properties, it is not surprising that SCFAs are also able to rescue BMECs from the effects of LPS *in vitro*. In monoculture conditions, propionate directly prevented LPS-induced tight junction disruption and also reduced levels of reactive oxygen species by inhibiting NRF2 in hCMEC/D3 endothelial cells.^12^ Propionate appears to exert these effects by binding to the G-protein coupled-receptor, free fatty acid receptor-3 (FFAR3) which is expressed by BMECs and to which propionate is a strong agonist.^37^

Knox et al. also demonstrated that both propionate and butyrate were protective against LPS-induced loss of barrier function (TEER) and promoted appropriate ZO-1 expression *in vitro*. A primary role of ZO-1 within the tight junction complex is to connect claudin-5 and occludin with the BMEC cytoskeleton, and SCFA supplementation in this instance increased the proportion of ZO-1 in contact with cytoskeletal actin filaments.^35^ Butyrate and propionate were also able to modulate the actin filament morphology, which is of interest as disorganisation of actin fibres and cytoskeletal architecture are a potential sign of cellular stress.^35,38^ As TEER readings were increased with SCFA treatment, this suggests the SCFA-induced F-actin remodelling was beneficial. However, further investigation is required to better understand the consequences for BMEC function after changes in actin structure. Furthermore, as there was no rescue of tight junction mRNA levels post LPS-treatment with either propionate or butyrate, it appears that the protective measures were not at the transcript level.^35^ This differs to other research findings, as it is understood that SCFAs, and butyrate in particular, are potent histone deacetylase (HDAC) inhibitors, promoting euchromatin and active transcription to increase protein levels of tight junctions, for example.^39^ Both butyrate and valproic acid (another SCFA) have been shown to exert beneficial effects in the context of BBB repair post-stroke via inhibition of HDAC and down-stream blockage of nuclear factor-kβ (NF-kβ) p65 transcription activity.^40^ This highlights that there is still significant knowledge to be gained regarding how SCFAs and other components of the gut microbiota secretome modulate BMEC structure and function on a molecular level, as it is likely that it is context dependent.

These promising results indicate benefits for SCFAs and the broader microbiota secretome in mediating BBB structure and function, and thus contributing to various neuropathologies (of which we have listed but a few, reviewed extensively by others^2,21,41^). However, the challenge remains regarding how to model these highly complex organ systems in order to understand the manner by which they interact and be able to modulate this to influence consequent pathophysiologies. *In vivo* animal models have been an extraordinary resource, identifying a fundamental requirement for a gut microbiota for appropriate BBB formation. In addition, these models can provide significant insight into the manner by which these numerous organ systems (i.e. the gut and the brain) interact simultaneously whilst allowing real-time cognitive-behavioural testing to determine clinically relevant outcomes. However, *in vivo* models generally require animal sacrifice prior to BBB readouts (to measure levels of stained dyes within the CNS) which necessitates either a large number of animals to determine time-course of pathology, which is both labour- and cost-intensive, or designing an experiment with a single outcome time-point which risks overlooking important earlier or later events in BBB pathology.^42^ Recent advances in *in vivo* imaging techniques look to address this limitation and provide a superior option for real-time dynamics of BBB permeability. However, these techniques require highly expensive imaging set-ups and lack standardisation as well as the sensitivity to identify less severe BBB disruption.^43,44^

*In vitro* models look to address these limitations by providing highly malleable experimental conditions allowing high resolution and repeated outcome measures to investigate cause-effect relationships. However, there is a clear trade-off here, with overall loss of physiological complexity necessitating highly specialised culture techniques and conditions (e.g. combining anaerobic and aerobic conditions for microbial co-culture with human cells), which attempt to recapitulate characteristics of *in vivo* conditions in a physiologically relevant manner. These seemingly incompatible model systems therefore create a dilemma in trying to characterise microbiota-BBB interactions to advance knowledge. Of the two approaches to modelling (*in vivo* vs *in vitro*) gut-*in vitro* microbiota and BBB interactions, *in vitro* methods have undergone more rapid and drastic advances in the last decade, relative to the previous approaches. As such, the remainder of this review will focus on describing these developments and identifying future areas for innovation.

## ***In vitro*** models of the blood-brain barrier which can be used to interrogate microbiota-brain interactions

*In vitro* models provide the ability to finely dissect molecular pathways of BBB damage or repair, as well as the potential efficacy of therapeutics. They also have the advantage of being relatively high-throughput and less labour and cost-intensive than complete organism models. With recent advances in stem cell modelling, it is also possible to produce models using human cells which are reasonably physiologically relevant (i.e. reproducing key structural and functional features of *in vivo* tissue), particularly by combining multiple cell types of the NVU or by utilising 3D microfluidic technology. In this section we will summarise the various assays and available models to characterise barrier function or cellular phenotype in the context of microbial disruption or intervention.

### Measures of blood-brain barrier permeability *in vitro*

To compare various methods of modelling the BBB, initially we need to discuss the available strategies/criteria for determining function, as various models incorporate or meet these criteria with varying fidelity (summarised in Table 1).

**Table 1.** Key outcome measures to assess *in vitro* BBB function.

Outcome measure	Description	Associated BMEC/BBB phenotype
Functional		
TEER	Measures the resistance to ion flow between two compartments, i.e., across a tissue barrier	A decrease in TEER is indicative of greater barrier permeability. *In vitro* TEER > 500 Ω/cm^2^ broadly considered relevant to *in vivo* physiology
Permeability of fluorescent tracers	Measure of paracellular permeability across a cellular barrier. Commonly used tracers include sodium fluorescein and FITC-dextran	Sodium fluorescein indicates small molecular permeability (MW 376 Da) while FITC-dextran as a larger tracer (MW 4 or 70 kDa) indicates greater changes in barrier permeability
Permeability of transcytosed substrates	Permeability of HRP or Cy3-tagged transferrin across the cellular barrier indicates the functionality of cellular trafficking via caveolae or receptor mediated transcytosis	Changes in BMEC transcytosis are context dependent, i.e., can be beneficial or detrimental
Efflux transporter activity	Pgp activity (key BMEC efflux transporter) can be assayed via measuring cellular accumulation of Rhodamine 123	Negligible substrate accumulation indicates normal Pgp activity
Structural		
Adherens junctions (CD144)	Detection and quantification at mRNA level (PCR) or protein level (immunofluorescent analysis). Visualisation at the protein level has the advantage of ensuring appropriate cellular localisation	CD144 (VE-cadherin) is a key marker of endothelial identity
Tight junctions (claudin-5, occludin, ZO-1)		Claudin-5 is considered the dominant tight junction protein of the BBB, while ZO-1 functions to connect claudin-5 and occludin to the cytoskeleton Appropriate membrane localisation is important for barrier function with cytoplasmic translocation resulting in increased barrier permeability
Expression of transporters		High expression of efflux transporters (P-gp, MRP, BCRP). Uptake transporters to meet CNS metabolic demands (GLUT1, MCT1, LAT1)
Caveolae (CAV-1)		Direction of change in CAV-1 expression is context dependent, i.e., can be beneficial or detrimental
Cytoskeletal morphology	Immunofluorescent analysis of F-actin fibres provides insight into cytoskeletal and cellular morphology	Formation of F-actin stress fibres (parallel morphology) is linked to translocation of adherens and tight junction proteins to the cytoplasm and endothelial swelling

TEER: trans-endothelial electrical resistance.

#### Functional measures

Central to the BBB’s core role is its ability to regulate transport of systemically circulating compounds into the brain, and hence, defining the functional attributes of any BBB model is critical. Most BBB models to date have used 2D transwell systems, in which BMECs are cultured on the apical membrane and permeability between the apical and basolateral compartments is regulated by the cellular monolayer.^45^ In this set-up, trans-endothelial electrical resistance (TEER) is commonly used to assess barrier integrity. TEER provides (predominantly) manual, repeated-measures of permeability across the monolayer, with readouts indicating the level of resistance to ion flow between electrodes inserted into the apical and basolateral compartments.^46^ Some novel 3D and microfluidic models have also developed innovative approaches to incorporating electrodes into devices to retain the opportunity for real-time read-outs of barrier function.^47^ A reduction in TEER is generally considered to indicate a decrease in barrier function; however, TEER values can also be influenced by cell number and size which in turn can be impacted by experimental conditions.^48^ A minimum TEER value of 500 Ω/cm^2^ is considered sufficient to restrict small drug transport in a manner which physiologically resembles human *in vivo* BBB functions.^49^

Translocation of fluorescent tracers across the cerebral endothelial monolayer is also routinely used to assess barrier function, in parallel to TEER, providing a more clinically relevant readout. The addition of the tracer at the commencement of the experimental window and repeated sampling of the ‘brain’ (basolateral) compartment of the model allows for quantification of permeability across multiple time-points. The most common fluorescent solutes used are sodium fluorescein (molecular weight 376 Da) and fluorescein isothiocyanate (FITC) labelled dextran (molecular weight 4 or 70 kDa).^50^ Sodium fluorescein is reminiscent of small molecular permeability and is therefore more useful for detecting subtle changes in barrier functionality. The presence of both sodium fluorescein and FITC-labelled dextran can be easily detected by a fluorescent plate reader, providing a sensitive and objective measure of paracellular permeability. Sucrose[^13^C12] (molecular weight 354 Da) is an alternate permeability marker which may be considered most appropriate due to its metabolic stability, lack of protein binding and non-radioactive nature (in comparison to the earlier iteration [^14^C] sucrose).^50^ However, to analyse levels of either sucrose isoform requires sensitive LS-MS/MS techniques and is therefore a less accessible option than fluorescent alternatives.

*In vitro* models of the BBB also allow for assessment of transcellular permeability. The predominant method for cerebral endothelial transcellular trafficking is via caveolae formation: a section of the membrane, enriched with a caveolin-1 protein sheet, which invaginates around the molecule to be trafficked. Horseradish peroxidase (HRP) is transcytosed via caveolae-dependent mechanisms, therefore injection of HRP to the apical surface of cerebral endothelial cells and consequent collection of basolateral medium, quantifies the volume of cellular transcytosis mediated by caveolae.^51^ A similar method can be used to quantify the alternative route of cellular trafficking – receptor mediated transcytosis – using Cy3-tagged transferrin.^51^ Another crucial component of transcellular permeability is the ability of BMECs to restrict entry via appropriate efflux transporter activity. P-glycoprotein (P-gp, ABCB1) is one of the specialised efflux transporters expressed by BMECs, and P-gp activity is generally used as a surrogate for overall efflux function *in vitro*. Rhodamine 123 is a P-gp substrate, and cellular accumulation of Rhodamine 123 (1-h incubation followed by cell lysis) can be quantified to determine P-gp activity, with negligible accumulation indicative of appropriate P-gp function.^52^

#### Structural measures

To perform the highly specialised functions of the BBB, BMECs must express the cellular structures responsible for performing these functions. Adherens junction protein, VE-cadherin (CD144), is an important marker of cerebral endothelial cell identity as interaction of VE-cadherin with cytoskeletal β-catenin, and has been shown to activate transcription of key tight junction protein – claudin-5.^53^ Claudin-5 is the most dominant BBB tight junction protein, significantly enriched in BMECs compared with other endothelial cells, although claudins-1, -3 and -12 are also found within tight junction complexes in the brain.^54^ Downregulation or delocalisation of claudin-5 from cell membranes results in tight junction disruption and consequent transcellular leakage of the BBB,^54^ hence enriched and localised claudin-5 is a strong indicator of a restrictive barrier. Occludin is another important protein of the tight junction complex, maintaining BMEC monolayer tightness and disruption of occludin expression has been linked to BBB extravasation in models of ischaemic stroke, Alzheimer’s disease and brain metastases.^55–57^ The third major protein of the tight junction complex is ZO-1, which functions to connect claudin-5 and occludin to cytoskeletal filaments, and similarly, inappropriate ZO-1 expression or configuration contributes to depleted barrier function via junctional leakage.^58^ Aberration in junctional staining is generally visualised via immunofluorescent staining for the junctional proteins of interest and confirming appropriate membrane localisation. This is often quantified at the mRNA level; however, this over-looks the importance of appropriate junctional formation and as such, may underestimate more subtle changes. Quantification of the homogeneity of immunofluorescent staining intensity along the junction, is a published alternative with greater spatial sensitivity.^59^

Intracellularly, the tight junction complex is highly regulated by the cytoskeleton, from establishment through to maintenance. In fact, in epithelial cells, direct disruption of actin fibre polymerisation is sufficient to interrupt tight junction organisation and function.^60^ Additionally, the cytoskeleton tightly controls changes to endothelial morphology in response to potentially damaging conditions, rearranging F-actin filaments into a parallel stress fibre orientation. For instance, F-actin stress fibre formation was more apparent in singularised endothelial cell cultures which completely lack cellular adhesion, compared with cell-cell adherent monolayers.^38^ Addition of a VE-cadherin antibody to endothelial monolayers also initiated the formation of F-actin stress fibres and resulted in a morphology of endothelial swelling.^61^ As such, it appears that F-actin filament arrangement is an important marker of cerebral endothelial barrier function. For example, hypoxia-hypoglycaemic conditions (*in vitro* model of ischaemic injury) rapidly upregulate the parallel F-actin formation in BMEC transwell cultures, which induced changes to normal endothelial morphology and redistribution of occludin and VE-cadherin from the membrane to the cytoplasm.^62^

BMEC transcellular permeability can also be interrogated at the structural level. ATP-binding cassette (ABC) transporters are highly enriched at physiological tissue barriers, such as the BBB.^63^ Three main ABC transporters groups, B, C, G, are particularly important for efflux transport, of which ABCB1 (multi-drug-resistant protein – MDR1, P-gp), ABCC (multi-drug resistance associated protein, MRP) and ABCG2 (breast cancer resistance protein, BCRP) are the most common transporters and their expression can be used to quantify BBB efflux.^63^ Uptake transporters for barrier trafficking are also important measures of barrier function, ensuring the BBB can deliver sufficient nutrients to the CNS to maintain neural homeostasis.^64^ Glucose transporters are particularly important to meet the high energy demands of the CNS, GLUT1 (SLC2A1) is the main glucose transporter expressed at the BBB.^65^ Other important transporters include MCT1 (monocarboxylate transporter, SLC16A1) and LAT1 (L amino acid transporters, SLC7A5) for lactate and amino acid uptake, respectively.^66,67^ Caveolae formation for vesicular transport can also be assessed via quantification of CAV-1 expression, either at mRNA or protein level.^68^ In some instances, caveolae formation is linked to increased barrier permeability, via the endocytosis of tight junctions, and therefore high CAV-1 expression can be detrimental.^68^

### Cellular and technical considerations for BBB modelling

**Figure 2.**
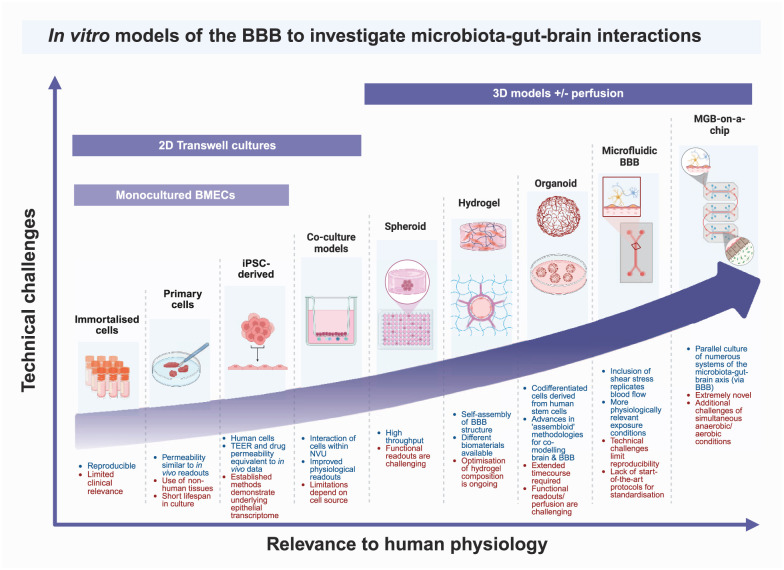
Comparison of the available options for developing *in vitro* BBB models. Original figure created with BioRender.com.

Key considerations summarised in Figure 2.

#### Monoculture models

The BBB can be modelled in a relatively simple manner using a variety of monoculture strategies, including immortalised, primary and induced pluripotent stem cells (iPSCs)-derived BMECs. The least labour intensive and most economical option is to utilise monocultured immortalised brain endothelial cells, with most common hCMEC/D3 or hBMEC cells. Immortalised cells are available from multiple species and can be cultured for high number of passages, with low batch effect or inter-experiment variability.^69^ When cultured on the apical membrane of transwells, these cells form a monolayer reminiscent of fundamental structure of the BBB.^69–73^ However, with immortalisation processes and extensive passage number, these cells lose the highly specialised cellular identity which BMECs require to perform their restrictive barrier function, with reduced tight junction protein expression and TEER values (<200 Ω) which are considered lower than minimum values required to restrict small molecule permeability in a manner similar to *in vivo* measures.^19,49,74^ Despite this, immortalised BMECs do respond to barrier damaging/reinforcing stimuli and hence, provide a high-throughput and reproducible approach to modelling barrier function.^35^

Primary BMECs from rodent or porcine sources can also be used to investigate BBB pathologies; however, primary cells are more labour intensive to collect and demonstrate phenotypic drift after at least three passages.^75–77^ Human-derived primary BMECs are obviously even harder to source, as healthy brain biopsies are collected extraordinarily rarely.^78^ Primary human BMECs are also available commercially but have the same limited lifespan in culture. Despite these logistical limitations, primary BMECs are much more physiologically relevant than immortalised cells, routinely producing TEER > 500 Ω/cm^2^ when cultured in transwells^75,76,79^ while primary (bovine) BMECs demonstrate restricted permeability of radiolabelled ligands comparable to permeability studies of the (anaesthetised) rat brain.^80^

More than a decade ago Lippmann et al. developed the first protocol by which to differentiate iPSCs to BMECs.^81^ This approach involves a neuroendothelial differentiation of iPSCs, and with the addition of retinoic acid in later stages of differentiation, this protocol has been widely characterised to produce TEER values > 1000 Ω/cm^2^, expression of tight junction complexes and high levels of efflux transporters, and very low paracellular permeability.^61,82–86^ In fact, a monolayer of iPSC-derived BMECs demonstrates a significant and strong correlation to *in vivo* human pharmacokinetic data of small drug permeability.^87^ Additionally, while direct comparison of electrical resistance with *in vivo* human data is clearly challenging, TEER recordings using glass microelectrodes of *in vivo* rat arterial and venous microvessels have been reported at 1490 and 918 Ω/cm^2^, respectively.^88^ These findings underscore the value of deriving BMECs from iPSCs, as this is the only source that reliably achieves TEER levels comparable to *in vivo* measures. However, numerous adaptions to the original protocol have been published, such as Wnt supplementation^89^ or differentiation under hypoxic conditions,^90^ with inter-batch or inter-iPSC-line variability in cell marker expression necessitating significant optimisation provided as the rationale for such adaptions.^91^ Additionally, the transcriptome of the derivatives of the original and commonly applied method is more epithelial in nature (lacking key endothelial-lineage genes). While the functional consequences of these transcriptomic limitations for barrier permeability remain unclear, Li et al. demonstrated an inability of these iPSC-derived ‘BMECs’ to form lumen structures *in vivo* which could be overcome by transduction of endothelial specific transcription factors.^92,93^

#### Co-culture models

As the NVU is comprised of multiple cell types, which provide physical and chemical support to the BMECs, *in vitro* models which incorporate additional cell types clearly possess greater physiological relevance. There are similar considerations for sourcing these co-cultured support cells, with immortalised more easily obtained and less labour- and cost-intensive, whilst primary or iPSC-derived known to be more biologically reliable.^11^ A further advantage of deriving BBB cells from iPSCs is the opportunity to generate not only patient-specific BMECs but also other NVU cell types from the same individual, a clear step towards precision medicine approaches to studying the interplay of the microbiota-gut-brain axis.^94^ These patient-derived, isogenic models have shed light on BBB pathologies associated with conditions such as cerebral cavernous malformations^95^ and psychomotor retardation^96^ and also offer significant potential for advancing our understanding of microbiota-BBB interactions in a highly clinically-relevant manner.

##### Astrocytes

Astrocytes exert significant control over the phenotype of brain endothelium, and as such, they are commonly co-cultured with BMECs to model the BBB *in vitro*. Astrocytes secrete factors, such as vascular endothelial growth factor (VEGF), glial cell line-derived neurotrophic factor (GDNF), basic fibroblast growth factor (bFGF) and angiopoietin-1, which promote and maintain the specialised BMEC phenotype.^97^ *In vitro* co-culture of primary BMECs from rodent and porcine sources with primary astrocytes of the matched species in transwells increased TEER values when compared to mono-cultured BMECs, suggesting increased resistance to paracellular permeability was achieved with astrocyte paracrine signalling.^98^ Astrocytic Wnt signalling seems to be highly influential upon transcellular BBB permeability, with co-culture of primary astrocytes from conditional Wnt knockout mice with primary BMECs, eliciting increased transcellular passage and caveolin-1 expression.^99^ Astrocytic end-feet are particularly important for BBB maintenance as they directly contact the BMEC basement membrane and help to surround the capillary *in vivo*. Direct contact of end-feet with BMECs *in vitro* by culturing the astrocytes on the basolateral surface of the transwell filter provides synergistic support producing elevated TEER in comparison to a non-contact co-culture approach.^100,101^ Novel models, such as microfluidic BBB-on-a-chip also allow for direct contact between BMECs and astrocytes, and hence, incorporate an additional level of synergy.^102^

Inclusion of astrocytes in models to investigate the BBB in the broader context of microbiota-brain interactions is particularly important as a number of microbial metabolites are capable of exerting significant influence over astrocytes. LPS promotes a pro-inflammatory phenotype in cultured astrocytes, inducing increased secretion of pro-inflammatory cytokines (TNFα, VCAM-1, IL-1β, IL-6, IL-15, IL-27).^103,104^ Conversely, SCFAs promote *in vitro* astrocyte expression of neurotrophic BDNF (in a sex-specific manner)^105^ and can alleviate stress (hypoxia)-induced primary rat astrocyte activation.^106^ With this susceptibility to microbial product-mediated effects and their important role as modulators of the BBB, it is likely that capillary-associated astrocytes regulate the effects of microbial products at the neurovascular unit. For example, LPS stimulation of BMEC monocultures resulted in lasting barrier disruption; however, the damage was transient in primary BMEC/astrocyte co-cultures, indicating an important role of astrocyte-mediated recovery.^107^ As such, *in vitro* models of the BBB which incorporate astrocytes provide not only greater physiological relevance at baseline but also in the context of exposure to circulating microbial products.

##### Pericytes

The role of pericytes to the NVU is less well-characterised but emerging evidence indicates their criticality to models of the BBB *in vitro*. In fact, co-culture of primary rat-derived BMECs with primary rat-derived pericytes induced 400% greater TEER recordings than co-culture with primary astrocytes.^108^ This was likely via upregulating tight junction proteins as pericyte conditioned medium increased occludin mRNA and protein levels in brain endothelial cells, predominantly via angiopoietin-1 signalling.^109^ Pericytes also regulate the transcellular permeability of BMECs – co-cultured pericytes enhanced P-gp efflux transporter function while concurrently inhibiting paracellular flux of sodium fluorescein, in a TGF-β-dependent manner.^110^ Pericytes are also sensitive to gut microbial influence^111^ with clear consequences for BBB function *in vitro*. Transcellular passage of radioactively-labelled HIV by LPS-stimulated primary BMECs was exacerbated with the addition of primary pericytes to the experimental design, seemingly linked to increased pericyte secretion of pro-inflammatory cytokines (G-CSF, GM-CSF, IL-1α, IL-6, IP-10, KC and MCP-1).^112^

##### Microglia

The importance of microglia to the neurovascular unit is yet to be fully understood, with the term ‘capillary-associated microglia’ a relatively new one,^113^ despite localisation of microglia to the vasculature being observed as early as 1972.^114^ Microglia have classically been considered to have two major phenotypical categories: resting/ramified or activated/amoeboid, with each phenotype inducing opposing effects on brain endothelial cells. However, emerging evidence indicates that microglial phenotypes exist along a continuum, with multiple intermediate and functionally diverse activation states.^115^ In their resting state, microglia have shown barrier promoting effects, upregulating ZO-1 and occludin expression in co-cultures.^116^ However, inducing a more activated phenotype of microglia with neurotoxic amyloid-β reversed these beneficial effects on tight junction formation and increased passage across the endothelial monolayer.^116^ As the resident immunocompetent cells of the CNS, microglia play a crucial role in relaying pathogenic microbial signalling to the neuronal microenvironment. As such, they are useful cells to include in *in vitro* models of BBB mediated microbiota-gut-brain axis communication.

Similarly to other cells of the NVU, microglia are highly susceptible to both pathogenic and beneficial influence of the microbial secretome. For instance, secretion of pro-inflammatory TNF-α from *ex vivo* cultured microglia from LPS-treated mice was partially reversed when the mice were also placed on a high soluble fibre diet (inulin) which increases plasma SCFA concentrations.^117^ Likewise, direct supplementation of microglia with SCFAs (butyrate and acetate, alone and concurrent) suppressed cytokine secretion.^117^ As pro-inflammatory cytokines are able to disrupt tight junctions,^118^ modulation of microglial cytokine release is likely to have consequences for BBB function. However, *in vitro* characterisation of microglial-endothelial dynamics in response to microbial-derived metabolites or products remains an under-researched area. This is a substantial knowledge gap as capillary-associated microglia are thought to play a considerable role in mediating cerebral blood vessel function, potentially through pericyte-dependent mechanisms.^113,119^ As such, further investigation is required to understand the molecular underpinnings of microglial influence over barrier permeability following gut microbial-directed intervention.

##### Neurons

The addition of neural-lineage cells to *in vitro* models of the BBB, allows the direct neurotoxic or neuroprotective effects of microbial-derived products on the BBB to be determined. In turn, addition of neural lineage cells enhances the biomimetic nature of the culture conditions (i.e. closely replicates our understanding of *in vivo* physiology) and promotes BMEC function. Primary rat neural progenitor cells (NPCs) enhanced BMEC structural and functional readouts (ZO-1 staining and TEER) in a transwell co-culture system.^120^ Other experimental models have demonstrated similar beneficial effect of NPCs or mature neurons on BMEC phenotypic and transcriptomic authenticity.^121^ Furthermore, addition of NPCs to astrocyte-BMEC cultures to form a tri-culture model has been shown to improve the astrocyte end-feet connection to the endothelial monolayer, emphasising the complex crosstalk between numerous cells of the NVU.^121^

Reciprocally, primary co-culture of endothelial cells with hippocampal NPCs promoted progenitor differentiation,^122^ indicating a mutually beneficial relationship between these cells *in vitro*. There are a limited number of published co-culture models of BMECs with mature neurons, probably associated with the financial and technical challenges of long-term neuronal differentiation. This is clearly a gap which should be addressed to improve the translatability of laboratory models to clinical outcomes as the postnatal brain is comprised predominantly of mature, quiescent neurons. In the few studies available, the co-culture of BMECs with mature neurons appeared to promote neuronal morphology and function^123^ and exert neuroprotective effects in the face of hypoxia^124^ and hyperglycaemia.^125^ Additionally, while there is some evidence to suggests that direct supplementation of monocultured neural lineage cells with SCFAs promotes neural differentiation and function,^126,127^ given the extensive crosstalk with other cells of the NVU, it is likely that SCFA effects upon neurons are also modulated by other cells more proximal to the metabolites in circulation (i.e. endothelial cells, astrocytes, pericytes). As such, inclusion of neural cells into models of the BBB designed to study the humoral pathway of the gut-brain axis would provide rich insight into direct mechanisms of influence.

#### 3D and microfluidic models

In addition to the complexity of the cellular components of the BBB models, increasing structural complexity also advances its biomimetic nature. 3D modelling of the BBB, which utilise novel biomaterials such as hydrogels, allow for the self-assembly of multiple cells of the NVU, to investigate how the entire system responds to stimuli. Seeding endothelial cells, pericytes and astrocytes into a biocompatible extracellular matrix leads to endothelial polarisation and formation of a tubular structure with a capillary lumen.^128^ Evaluation of this 3D model determined enhanced claudin-5 expression relative to a 2D transwell comparator and functionality was validated by increased permeability following exposure to the hyperosmolar agent – D-Mannitol.^128^

3D models can also be developed in a spheroid formation by seeding NVU cells into low attachment, cell repellent plates. NVU cells will then self-assemble with an astrocytic core, an outer monolayer of endothelial cells in direct contact with sub-located pericytes.^129,130^ Similar to the hydrogel method, increased expression of tight junction proteins and efflux transporters is exhibited with the spheroid formation versus a 2D configuration.^129^ Paracellular permeability can be semi-quantified in this model via analysis of a fluorescent tracer within the core and fluorescent intensity of Z-stack images analysed; however, full quantification of these methods has not yet been achieved.^131^ While this spheroid structure is less physiologically accurate than the tubular structure formed within the hydrogel, the spheroid model can be more easily and reproducibly upscaled, with Simonneau et al. producing >3000 spheroids/experiment.^130^ However, to date these models have primarily used immortalised human cells or primary cells from model species, and thus, they are undermined by the associated limitations of each.

Organoid structures which utilise human stem cells (embryonic or induced) have also been published; however, developing a vascularised organoid system is more challenging and labour-intensive than the hydrogel or spheroid alternatives. Long-term culture of cerebral organoids with VEGF and Wnt7a or specific transcription factors does eventuate in vascularisation^23,132^; however, the experimental time-course for this (30-70 days) is considerably >~7 days of spheroid or hydrogel approaches. Despite the more laborious protocol, given that all cells are derived from co-differentiated human stem cells (endothelial cells, astrocytes, pericytes, neural lineage cells all observed)^23,132^ they serve as a relevant recapitulation of the human NVU in entirety. In addition, newly emerging approaches termed ‘assembloids’ allow brain organoids and blood vessel organoids to be generated separately and subsequently combined to create BBB assembloids.^95^ These BBB assembloids demonstrate extensive NVU crosstalk, and patient-derived constructs have already been used to characterise novel mechanisms underlying inherited cerebrovascular disease.^95^ However, organoids are also not without limitation, structurally they are similar to spheroids with a less physiologically relevant cellular organisation and are inherently difficult to perfuse (presenting with challenges for permeability measures).^133^ Organoids also present with challenges regarding oxygen and nutrient delivery to the core, which, when considered in light of other limitations (e.g. extended time-course), appear to discourage many researchers from using cerebral organoid-based approaches to BBB modelling.

Many approaches to modelling the BBB three-dimensionally now also incorporate the ability to perfuse such a device (also termed BBB-on-a-chip) with culture media used to recapitulate *in vivo* blood flow. Physiologically, BMECs are constantly exposed to a low level of shear stress (published estimates of 5-23 dyne/cm^2^).^134^ This is vastly different to the static conditions of usual cell culture approaches. In fact, endothelial cells cultured with shear force conditions exhibit reduced signs of oxidative stress and barrier pearmeability.^135^ This is in addition to the upregulation of tight junction proteins and efflux transporters observed in dynamic versus static conditions.^14^ These changes in barrier functionality are mediated by VE-cadherin, as the junctional protein is able to transduce mechanical stimulation from blood flow (or perfused media) to chemical signalling within the endothelial cell.^136^ Furthermore, in the context of investigating the interaction between circulating microbial metabolites and cerebral endothelium, there is an appreciable difference in the type of exposure modelled within a microfluidic device versus a static approach. Shear stress directly regulates the uptake of solutes by the endothelial monolayer,^137^ hence perfusion of a molecule of interest over the apical surface creates vastly different exposure conditions (which are more physiologically relevant) than a stagnant incubation period in static models.

As perhaps the most physiologically relevant approach to *in vitro* modelling of the BBB, microfluidic models are also the most labour intensive, expensive (e.g. requiring high cell numbers), and least reproducible (high batch variability).^138^ Additionally, there is a lack of standardisation between different microfluidic BBB models, with considerations spanning from materials and dimensions used for microfluidic device fabrication and composition of the basement membrane, to components of the ECM, shear stress flow rates, and ways to incorporate outcome measures within the device, impacting inter-study comparisons.^47^ Additionally, the significant complexity of these models increases opportunity for human error,^138^ reinforcing the low-throughput nature of this approach and reducing reproducibility across experimental set-ups. This is further exacerbated by the lack of consensus on state-of-the-art protocols. However, in terms of modelling the microbiota-gut-brain axis in entirety, combining multiple organ system models (1, brain-on-a-chip; 2, BBB-on-a-chip; 3, immune system-on-a-chip; 4, gut-on-a-chip; 5, microbiota-on-a-chip) to develop a microbiota-gut-brain axis-on-a-chip, microfluidic 3D modelling is clearly the most promising route.^139^ As such, efforts to improve microfluidic modelling of the BBB may revolutionise not only our approaches of modelling the microbiota-gut-brain axis but also our understanding of its role in health and disease.

## Bridging the ***in vivo***/***in vitro*** divide: ***In vitro*** models of the microbiota-gut-brain axis

Although advances in 3D, multi-cellular, and/or microfluidic modelling of the BBB are highly useful to study how the gut microbiota can reach the CNS via modulating this interface, these models still differ substantially to the *in vivo* environment. *In vivo* there are opportunities to investigate microbial changes with time (i.e. across the lifespan) or with progression of disease, as well as a straightforward approach to investigating multiple organ systems simultaneously (e.g. the influence of the immune system or the peripheral nervous system). Nonetheless, steps are being made to incorporate components of the microbiota-gut-brain axis into BBB models, and this section of the review will focus on these developments.

The most common *in vitro* approach for investigating gut microbiota-brain interactions via the BBB, involves applying commercially sourced gut microbial metabolites or products to cultured BMECs, often alongside other NVU cells. For example, stimulating BMECs with LPS and/or SCFAs to induce barrier deleterious or protective conditions, respectively.^12,35,140^ Other metabolites which have been investigated in this manner include: (i) trimethylamine N-oxide (TMAO), which enhances BBB integrity,^141^ (ii) *p*-cresol glucuronide (pCG), which prevents LPS-induced BBB damage^142^ and (iii) secondary bile salts, which increase BBB permeability.^143^ Other gut-derived compounds have also gained increasing research attention, including (poly)phenols and indoles, suggesting they also may exert BBB-modulatory effects and permeate the barrier to influence central neurophysiology.^144–146^ It is important that further research is performed to capture the importance of these compounds in the humoral pathway of the microbiota-gut-brain axis. While the method of applying gut-derived compounds to *in vitro* BBB models certainly aids in identifying the therapeutic potential of individual components, it is also a relatively simplified representation of the microbiota-gut-brain axis.

An alternative option involves exposing *in vitro* BBB models to cell-free microbial supernatants, that is, the entire microbial secretome. Recently, this approach was used with supernatants generated from *Bifidobacterium* following bacterial fermentation of infant formula, and this supernatant was able to prevent LPS-induced barrier permeability.^147^ While this method better captures the overall impact of microbial metabolites, it has been utilised more readily to investigate localised effects of gut microbes upon the gut epithelial barrier.^148^ Given the role of the humoral pathway in transmitting microbial signals to the CNS, this method may shed light on how microbial disruption or beneficial microbial profiles influence BBB integrity in CNS disorders. An extension of this approach is the exposure of BBB models to *ex vivo* plasma. With the added benefit of incorporating host immune factors (e.g. pro-inflammatory cytokines), this method provides the most physiologically relevant representation of the milieu to which endothelial cells (including those of the BBB) are exposed. While yet to be applied to microbiota-gut-brain axis research, this method has been successfully applied in other contexts, such as proof-of-concept microfluidic modelling^102^ or to investigate COVID-19 related changes to BBB permeability.^149^

Another potential avenue is the direct co-culture of microbes with NVU cells. Previously used in CNS infection models,^150^ this method faces challenges due incompatible gaseous demands – aerobic and anaerobic conditions for BMECs and microbes, respectively. However, the Human oxygen-Bacteria anaerobic (HoxBan) co-culturing system is an innovative set-up which allows the co-culture of anaerobes with human cells by growing microbes in solid agar, creating mutually optimal conditions.^151^ Although direct interaction between gut microbes and NVU cells typically requires severe gut barrier compromise – limiting its clinical relevance for most contexts – this method illustrates how technological innovation is expanding the capacity to model inter-organ system interactions *in vitro*.

Multi-organ devices which can replicate multiple components of microbiota-gut-brain axis interaction are emerging. This is clearly a step beyond the once siloed approaches of *in vitro* models and a step towards *in vivo* physiology, with the added benefit of greater experimental control and the use of human tissues. While still a very novel area of research, the emerging models have provided rich insight into the interactions of these physiological systems (summarised in Table 2). These models involve innovative approaches to modelling the microbial influence on the brain, either via direct co-culture of microbial components^152,153^ or via isolated components (i.e. metabolites, exosomes) or bacterial conditioned medium.^153–156^ They can also provide insight into direct cause-effect relationships for gut-derived brain pathology. For example, a model developed by Cho et al. indicates that conditioned medium from *Helicobacter pylori* induces gut barrier damage with subsequent BBB damage, and this permits greater entrance of endotoxin to the CNS.^155^ Additionally, by modulating the brain compartment to replicate neurodegenerative conditions – either by inducing amyloid-β accumulation via transduction of Alzheimer’s disease mutations or by delivering the pathological proteins which drive Parkinson’s disease (α-synuclein fibrils) – this model was able to determine that in CNS pathologies the BBB also delivers damage to the periphery.^155^

**Table 2.** Experimental approaches for integrated gut microbiota and blood-brain barrier modelling.

Study	Structure/approach	Microbiota component	Gut component	BBB component	Brain component	Additional innovation	Key findings
Kim et al. (2021)	Microfluidic chip (two interconnected channels)	Exposure to LPS or butyrate to model microbiota secretome	Caco-2 gut epithelial cells seeded in one channel	Primary human BMECs seeded in second channel	N/A	Investigated transport of gut-derived exosomes across BBB under static vs flow condition	LPS: ↓ TEER ↑ FITC permeability (gut and BBB) Butyrate: ↑ TEER ↓ FITC permeability (gut and BBB) Greater exosome uptake by BMECs with perfusion (vs static)
Mao et al. (2023)	Microfluidic chip with two chambers (first chamber: microbiota/gut; second chamber: brain) separated by a synthetic barrier	*E. coli* (K-12 strain) cultured in first chamber	N/A However, the neurotransmitters serotonin and dopamine can be gut-derived	Synthetic barrier: PEG gel which filled the micropores within the barrier; permeability manipulated by PEG composition	AHPC (rat-derived) neurospheres	Inbuilt electrochemical sensors allowed monitoring of serotonin and dopamine concentrations within the first (microbiota/gut) chamber compared with the second (brain) chamber; authors discussed intentions to include BMECs and astrocytes within the micropores in future work to further recapitulate human physiology	Neurotransmitters but not *E. coli* could transverse the synthetic barrier to influence AHPC neurosphere physiology (viability, proliferation and neurogenesis)
Modasia et al. (2023)	Static three-cell transwell model with gut epithelium cultured on the apical filter surface, brain endothelium cultured on basolateral filter surface, and CNS cells cultured in the basolateral compartment	Extracellular vesicles isolated from *Bacteroides thetaiotaomicron*	Caco-2 gut epithelial cells	Human hCMEC/D3 cell line	SH-SY5Y non-differentiated neuronal cells SIM-A9 microglia cultured in second model – hCMEC/D3, SH-SY5Y tri-culture	Inclusion of microglia for neuroimmune interactions	Bacterial-derived extracellular vesicles could permeate gut and BBB barriers (sequentially) and were then taken up by neural lineage cells
Kim et al. (2024)	Two separate microfluidic chips (first: gut-chip; second: brain-chip). Brain-chip exposed to supernatant or bacterial exosomes from gut-chip	Commercially available probiotics: *Lactobacillus casei* Hy2782 and *Lactobacillus plantarum* Hy7714 . Cultured within gut-on-a-chip	Caco-2 gut epithelial cells with a gastrointestinal lumen organisation	N/A	Human iPSC-derived neurospheres	Brain-chip had two compartments; second compartment allowed for neurite outgrowth to be measured	Metabolites and/or exosomes from probiotics promoted neural differentiation Metabolites and/or exosomes could partially alleviate some of the impaired neurite growth induced by prior Aβ stimulation
Cho et al. (2025, pre-print)	Microfluidic microbiota-gut-brain axis on a chip: three subsequently perfused channels: gut → BBB → brain or brain → BBB → gut.	*H. pylor*i (NCTC 11637 strain) cultured separately and conditioned media collected. LPS used as positive control	Caco-2 gut epithelial cells exposed to either *H. pylori* conditioned media or LPS	Human embryonic stem cell-derived BMECs	Human NPCs differentiated into neurons and astrocytes (within microfluidic chip for 21 days) Neurodegeneration modelled by NPC transfection with APPSL lentivirus (to induced familial Alzheimer’s mutations) or addition of α-synuclein fibrils (Parkinson’s pathology)	Bidirectional perfusion allowed for investigation of effects of neurodegeneration upon the gut – i.e., both bottom-up and top-down influence	Both bacterial conditioned medium and LPS induced gut and BBB barrier damage leading to increased endotoxin concentrations in brain compartment. Induction of Alzheimer’s model in brain compartment reduced synaptic connections and disrupted BBB tight junctions and gut villi α-synuclein fibrils also disrupted neural synapses and were translocated from brain through both BBB and gut barriers to gastrointestinal lumen.

AHPC: adult hippocampal neuroprogenitor cell; *E. coli: Escherichia coli; H. pylori: Helicobacter pylori*; PEG: poly (ethylene glycole).

While the biotechnology to achieve the goal of a complete microbiota-gut-brain axis model *in vitro* is still currently under active development, the MINERVA project has received European Research Council (ERC) funding to develop the first microbiota-gut-brain axis microfluidic device.^139^ This proposed device will incorporate communication and interaction of five of the involved systems: brain, BBB, immune, gut and gut microbiota within a single platform.^139^ This model will be used to investigate microbiota-gut-brain axis dysfunction in Alzheimer’s disease, with plans to use microbiota samples collected from Alzheimer’s patients compared to healthy controls.^139^ Other approaches under development include using a microbiota-gut-brain axis on a microfluidic chip to gain insights into neurodegenerative diseases led by Broersen at the University of Twente, the Netherlands.^157^ Moysidou et al. in University of Cambridge have also received ERC funding to develop a 3D bioelectronic model of gut-brain signalling.^158^ Finally, Sedrani et al. have developed neuroHuMiX which is an advanced gut-on-a-chip model to study the interactions of bacterial, epithelial and neuronal cells.^159^ This model allows unravelling of the molecular mechanisms underlying the communication between the gut microbiome and the nervous system. As with many such microbiota-gut-brain axis models it doesn’t as yet incorporate the BBB but serves as a template for further innovation in the space. While substantial progress has been made in advancing the technical resources and models available to study microbiota-BBB interactions, a significant gap in the research landscape persists – despite identification of microbial influence on the BBB, the underlying mechanisms of this influence continue to be relatively unexplored. As such, the field would benefit from leveraging these emerging models to study these interactions with a stronger focus on mechanistic aspects.

Despite these efforts to bridge the *in vivo*/*in vitro* divide to microbiota-BBB modelling, clear differences persist. As current models show, no cell culture system has yet integrated all five interacting organ systems which comprise the microbiota-gut-brain axis. Consequently, the reported effects of the microbial secretome on the BBB *in vitro* remain an approximation of true physiology. Nevertheless, the physiologically relevant read-outs demonstrated by existing BBB models (e.g. TEER and profiles of small drug permeability within *in vivo* ranges), coupled with the opportunity to incorporate a precision medicine approach by utilising iPSC technology to develop patient-derived BBB models, highlights the value of *in vitro* systems for fundamental insight, that is, higher-throughput secretome screens. In parallel, the importance of ongoing *in vivo* experimentation to assess influence of microbial BBB modulation on cognitive behavioural outcomes and inter-system interactions remains essential.

## Conclusions and future recommendations

Extensive research progress has been made to (i) uncover the interactions between the gut microbiota and its influence over the CNS and (ii) develop physiologically relevant models of the BBB *in vitro*. There is now clear evidence that the gut microbiota is aetiologically involved in neurological disorders, with an important role of the BBB to mediate these effects. As the gut microbiota can be modulated with relative ease, these interactions may provide insight into adjunctive or even disease altering interventions by targeting the methods by which the microbiota communicates with the CNS (such as the secretome). Therefore, it is also crucial that we ensure that *in vitro* models are able to provide the insight required in order to define these interactions, particularly via the BBB, which we know is instrumental in defining peripheral to central influence. Despite significant advancement, ongoing considerations for improving *in vitro* BBB (and microbiota-gut-brain axis) modelling to more faithfully replicate *in vivo* conditions remain. Biological factors which strongly influence both the BBB and gut microbiota such as sex^160^ and age^21^ which are modelled with ease in animals impose further challenges to *in vitro* set-ups and are often overlooked in experimental design. It is also important to consider that other CNS barriers, that is, the blood-spinal cord barrier and the blood-cerebrospinal fluid barrier, have received less research development attention than the BBB but are also significant mediators of CNS communication with the periphery, thus the field would benefit from improved modelling of these interfaces. With the significant advances in the manner by which we model not only the BBB, but also other organs, *in vitro*, it is now time for these research areas to work in concert to fully understand the mechanisms of microbiota-gut-brain axis communication and harness its potential in health and disease.
